# Variation of the Vaginal Microbiome During and After Pregnancy in Chinese Women

**DOI:** 10.1016/j.gpb.2021.08.013

**Published:** 2022-01-28

**Authors:** Xiaoai Zhang, Qingzhi Zhai, Jinfeng Wang, Xiuling Ma, Bo Xing, Hang Fan, Zhiying Gao, Fangqing Zhao, Wei Liu

**Affiliations:** 1 State Key Laboratory of Pathogen and Biosecurity, Beijing Institute of Microbiology and Epidemiology, Beijing 100071, China; 2 Department of Obstetrics and Gynecology, First Medical Center, The General Hospital of the People’s Liberation Army, Beijing 100853, China; 3 Computational Genomics Lab, Beijing Institutes of Life Science, Chinese Academy of Sciences, Beijing 100101, China; 4 Beijing Key Laboratory of Vector Borne and Natural Focus Infectious Disease, Beijing 100071, China; 5 Center for Excellence in Animal Evolution and Genetics, Chinese Academy of Sciences, Kunming 650223, China; 6 Key Laboratory of Systems Biology, Hangzhou Institute for Advanced Study, University of Chinese Academy of Sciences, Chinese Academy of Sciences, Hangzhou 310024, China; 7 University of Chinese Academy of Sciences, Beijing 100049, China

**Keywords:** Chinese pregnant women, Vaginal microbiota, Postpartum, Pregnancy complication, Premature rupture of membranes, Preterm birth

## Abstract

A comprehensive profiling of the vaginal microbial communities and their variability enables an accurate description of the microbiome in women. However, there is a lack of studies available on Chinese women. In the present study, the composition of the **vaginal microbiota** during pregnancy and the 6-week **postpartum** period of 454 Chinese women was characterized by sequencing the V3–V4 region of the 16S ribosomal RNA (rRNA) gene. The vaginal microbiome showed variations during pregnancy and the postpartum period based on the abortion history, hypertensive disorders, delivery mode, and maternal age. Co-variation of 22 bacterial taxa, including the *Lactobacillus* genus and two of its species, may account for the common characteristics of the vaginal microbiome under scenarios of different medical histories and pregnancy outcomes. In contrast, discriminant bacterial species were significantly different between women who had **preterm birth** (PTB) with and without **premature rupture of membranes** (PROM), and the community state type (CST) IV-A without any predominant *Lactobacillus* species in the microbiota was more prevalent during pregnancy in the PROM-PTB cases, suggesting that specific bacterial species could be considered to distinguish between different types of PTB. By providing data on Chinese women, this study will enrich the knowledge of the human microbiome and contribute to a better understanding of the association between the vaginal microbiome and reproductive health.

## Introduction

The Human Microbiome Project (HMP) reported and published human microbiome data derived from a myriad individuals [1]. As an important part of the HMP and one of the most prosperous communities in the human body, the vaginal microbiome has received considerable attention [2], [3]. In recent years, evidence is accumulating that vaginal microbiome is key to women’s health and a healthy pregnancy, and there is a gradual realization that the healthy status largely depends on whether this microbial community harbors more beneficial commensals or pathogens [4], [5], [6], [7], [8], [9]. Most studies have introduced information on the compositions and shifts in the vaginal microbiome in women belonging to African, European, and American ethnicities [10], [11]; however, there is a lack of research on the vaginal microbiome present in Chinese pregnant women.

In China, nearly half of the births were delivered via cesarean section in 2007–2008, and the rate was close to 60% in some cities [12], [13], [14]. With the commencement of the two-child policy in China in 2015, healthcare providers have persistently encountered greater challenges regarding a higher cesarean section rate, or an advanced maternal age, or both. Additionally, in China, more than 1 million preterm infants are born per year, with this number being surpassed only in India [15], [16], [17]. All such serious obstetrics- and gynecology-related issues have gained prominence as major public health concerns in China and worldwide. Previous studies have shown that the composition of the vaginal microbiome is associated with pregnancy, delivery mode, maternal age, and preterm birth (PTB) [4], [5], [6], [7], [8]. A comprehensive and complete characterization of the vaginal microbiome and its variability with pregnancy, delivery mode, or specific characteristics of pregnant women will enable an accurate diagnosis of women who possess an abnormal vaginal microbiome.

In recent years, our understanding of the vaginal microbiome has broadened with the performance of cultivation-independent high-throughput sequencing. Ravel et al. [18] performed a 16S rRNA gene survey of vaginal samples obtained from 396 North American women of four ethnic groups. They divided the vaginal microbiome into five community state types (CSTs) based on the dominant bacterial species. CST I, CST II, CST III, and CST V were found to be dominated by *Lactobacillus crispatus*, *Lactobacillus gasseri*, *Lactobacillus iners*, and *Lactobacillus jensenii*, respectively, and CST IV was defined as a lack of *Lactobacillus* spp. and comprised a diverse set of strict and facultative anaerobes. Following a longitudinal study in Western reproductive-aged women over a period of 16 weeks, it was suggested that the CSTs were dynamic in some women but were relatively stable in others [19]. Thereafter, CSTs have been widely used in studies conducted on the association between the vaginal microbiome and reproductive health for their effective handling.

Thus far, some studies linking the vaginal microbiome to PTB have yielded mixed, and even discordant, results [4], [5], [6], [7]. Romero et al. [4] did not observe any association between the vaginal microbiome and either the premature rupture of membranes (PROM)-related or non-PROM-related PTB in a predominantly African American cohort. One study reported that CST V was associated with clinically heterogeneous PTB in two predominantly Caucasian populations [5]. Lindsay et al. [6] detected and reported a significant positive association between CST III and non-PROM-related PTB in high-risk Caucasian, Asian, and African pregnant women. Recently, Digiulio et al. [7] replicated their previously reported associations [5] between a lower abundance of *Lactobacillus* or a higher abundance of *Garderella* and clinically heterogeneous PTB in a low-risk Stanford cohort. However, their previously hypothesized association [5] between PTB and a higher abundance of *Ureaplasma* was not replicated in the subsequent study [7]. Resolution of the mixed, and even discordant, findings of prior studies requires the investigation of a microbiome with different cases of PTB and PROM simultaneously in a study with a more comprehensive design and a larger cohort size.

In the present study, by performing 16S rRNA gene sequencing, we characterized and compared the vaginal microbiome communities of Chinese pregnant women according to multiple factors, including delivery mode, maternal age, abortion history, pre-pregnancy maternal weight status, and pregnancy complications, between pregnancy and the postpartum period. Furthermore, the relationships between the vaginal microbiome and the clinical features of adverse pregnancy outcomes, particularly PTB, were analyzed. We aimed to expand the current understanding of the vaginal microbiome of Chinese pregnant women and to assess whether the microbial community shifts over time or under certain conditions.

## Results

From July to December 2016, a total of 474 pregnant Chinese women attending the Department of Obstetrics at 301 Hospital (Beijing, China) for regular check-ups were enrolled in this study. Of the 474 volunteers recruited, 20 pregnant women were excluded because of medical complications (of the fetuses or pregnant women) necessitating the conduction of labor induction. Of the 454 women included in the final analyses, the vaginal swabs of 356 pregnant women were collected during pregnancy, and those of 98 women were collected at 6 weeks postpartum (Table S1). Using 16S rRNA-based sequencing, we obtained a total of 27,171,551 high-quality sequences from the vaginal samples derived from Chinese pregnant women, with a median of 59,912 and an interquartile range (IQR) of 57,311–62,351 sequences per sample. A total of 6343 operational taxonomic units (OTUs) were generated with a sequence similarity ≥ 97%.

### The vaginal microbial community shifts significantly from pregnancy to the postpartum period

**Figure 1:**
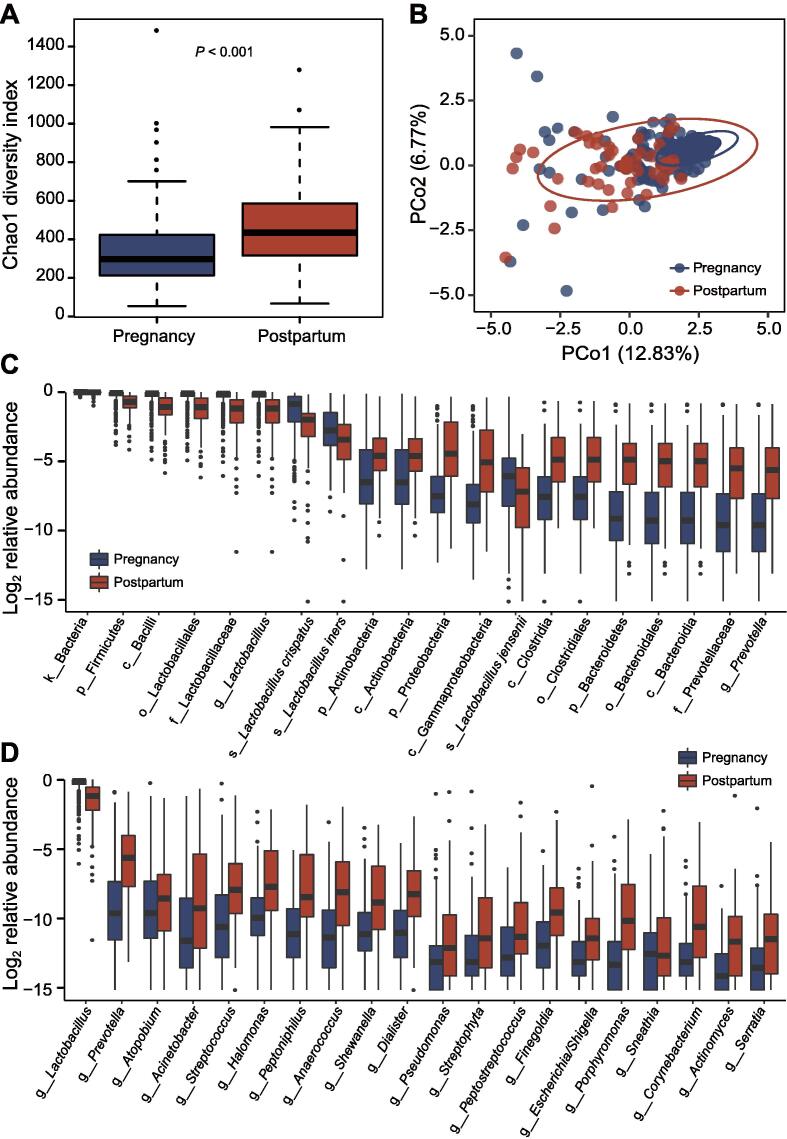
**The vaginal microbiomes during pregnancy and the postpartum period are significantly different in terms of the microbial diversity, community structure, and composition** **A.** Alpha diversity of the vaginal microbiomes between pregnancy and the postpartum period. Each box plot represents the median and IQR. The whiskers on each side of the box extend to the minimum or maximum value that is not an outlier. The circles beyond the whiskers represent outliers (> 1.5-fold IQR). **B.** Weighted ANOSIM and PCoA of the vaginal microbiomes between pregnancy and the postpartum period based on the distance matrix of UniFrac dissimilarity. The X- and Y-axes represent two dimensions explaining the most considerable proportion of variance in the communities. Each dot represents a sample, and each circle indicates a 95% confidence interval. **C.** Relative abundances of the significantly different taxa between pregnancy and the postpartum period at all levels. **D.** Relative abundances of the significantly different taxa between pregnancy and the postpartum period at the genus level. The discriminating taxa were identified based on the results of LEfSe analysis with a threshold of LDA score (log_10_) > 2 and *P* < 0.05. The prefixes k_, p_, c_, o_, f_, g_, and s_ represent the kingdom, phylum, class, order, family, genus, and species, respectively. IQR, interquartile range; ANOSIM, analysis of similarities; PCoA, principal coordinate analysis; LEfSe, linear discriminant analysis effect size; LDA, linear discriminant analysis.

**Supplementary Figure S1:**
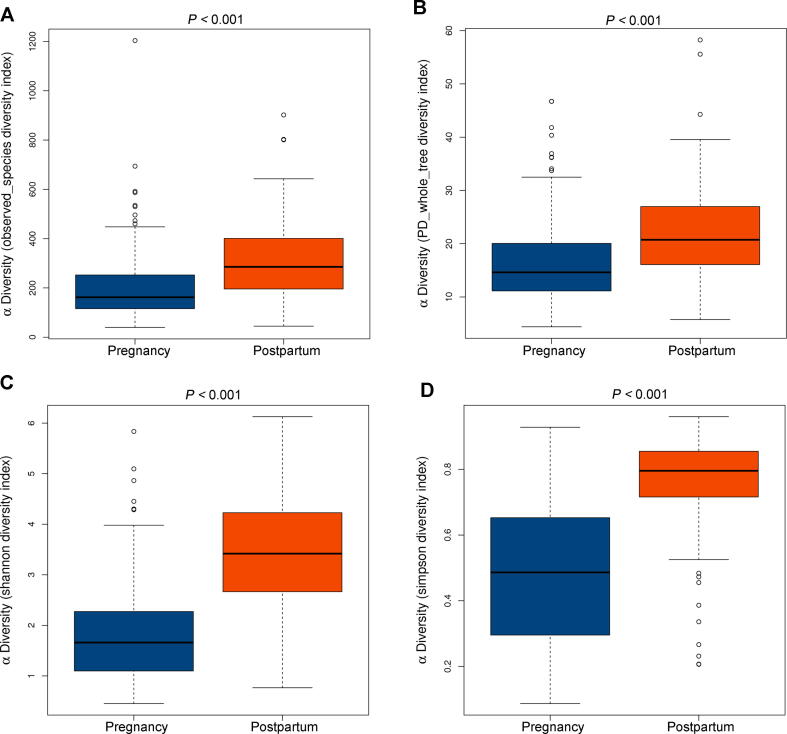
**The α diversities of the vaginal microbiome during pregnancy were significantly lower than those of postpartum** Differences in the α diversities of the vaginal microbiome between pregnancy and postpartum according to the observed species, PD whole tree (Faith’s Phylogenetic Diversity, which adds up all the branch lengths of the phylogenetic tree as a measure of diversity), Shannon, and Simpson index. Each box plot represents the median and interquartile range. The whiskers on each side of the box extend to the minimum or maximum value that is not an outlier. The circles beyond the whiskers represent outliers (>1.5 times interquartile range).

**Supplementary Figure S2:**
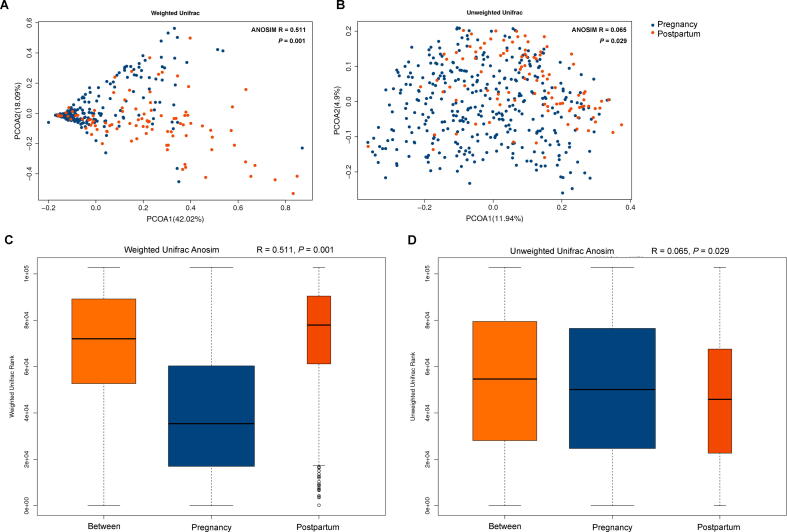
**The vaginal microbiome of pregnant women was more similar to each other during pregnancy** Weighted and unweighted PCoA (A & B) and ANOSIM (C & D) based on the distance matrix of UniFrac dissimilarity of the vaginal microbial communities in pregnancy and postpartum. Respective ANOSIM R values show the community variation between the compared groups, and significant P values are indicated. The axes represent the two dimensions explaining the greatest proportion of variance in the communities. Each dot represents a sample.

The α diversity quantified using the Chao1 index was significantly higher (*P* < 0.001) in the vaginal microbiome during the postpartum period than that during pregnancy (Figure 1A). The other four diversity indices showed consistent results (Figure S1A–D), indicating that the microbial richness might have increased markedly after delivery. Similarly, significant differences in β diversity were also observed based on the weighted UniFrac dissimilarities [analysis of similarities (ANOSIM) *R* = 0.511, *P* = 0.001] between pregnancy and the postpartum period (Figure 1B, Figure S2A–D). Moreover, the vaginal samples obtained from pregnant women were more closely clustered.

For each taxonomic category, the differences were substantial. At the phylum level, only the relative abundance of Firmicutes was higher during pregnancy than that during the postpartum period, while the abundances of Actinobacteria, Proteobacteria, Bacteroidetes, and other seven phyla were higher in postpartum women (Figure 1C). The *Lactobacillus* genus, as well as five of its species (*L. crispatus*, *L. gasseri*, *L. iners*, *L. jensenii*, and *Lactobacillus reuteri*), was significantly more abundant, and several genera, such as *Prevotella*, *Atopobium*, *Acinetobacter*, and *Sneathia*, were significantly less abundant during pregnancy (Figure 1D; Table S2).

**Supplementary Figure S3:**
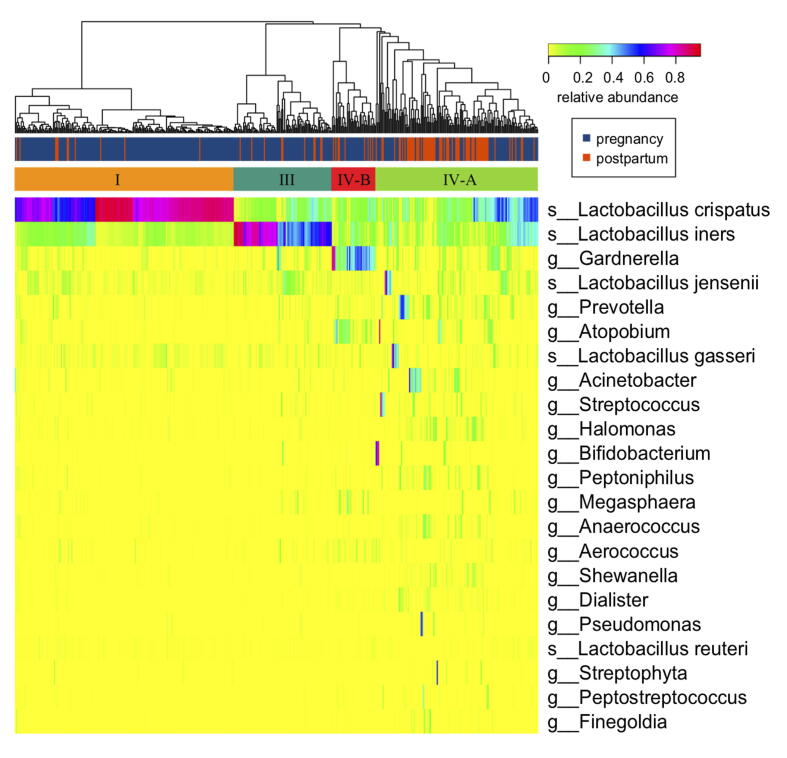
**The vaginal microbiome of most women after delivery was classified as CST IV-A** Color bar from yellow to red shows the relative abundance of each microbial taxon. The prefixes g_ and s_ represent genus and species, respectively. CST, community state type.

Considering an evident difference in *Lactobacillus* abundance before and after birth, we identified four CSTs (I, III, IV-A, and IV-B) in the vaginal microbiome of Chinese pregnant women (Figure S3; Table S3). CST I and CST III were dominated by *L. crispatus* and *L. iners*, respectively. Both CST IV-A and CST IV-B were dominated by non-*Lactobacillus* species, and the difference between them was that some women in the former continued to present with a certain proportion of *L. crispatus*, *L. iners*, *L. jensenii*, and *L. gasseri* in their microbiome, while the latter contained a higher abundance of *Gardnerella*. The most prevalent CSTs observed in Chinese pregnant women were CST I (41.9%), followed by CST IV-A (31.1%), CST III (18.7%), and CST IV-B (8.3%). The samples obtained from pregnant women were distributed throughout all types of CSTs; however, they were mainly identified to be CST I and CST III. In contrast, the postpartum samples were observed to be in CST IV, especially those with greater numbers of CST IV-A (*χ*^2^ = 92.08, *P* < 0.001). These results indicate that, in Chinese women, the vaginal microbiome lacks a certain proportion of *Lactobacillus* after delivery, which increases the microbial diversity; however, it is not completely dominated by harmful bacterial members such as *Gardnerella*.

**Supplementary Figure S4:**
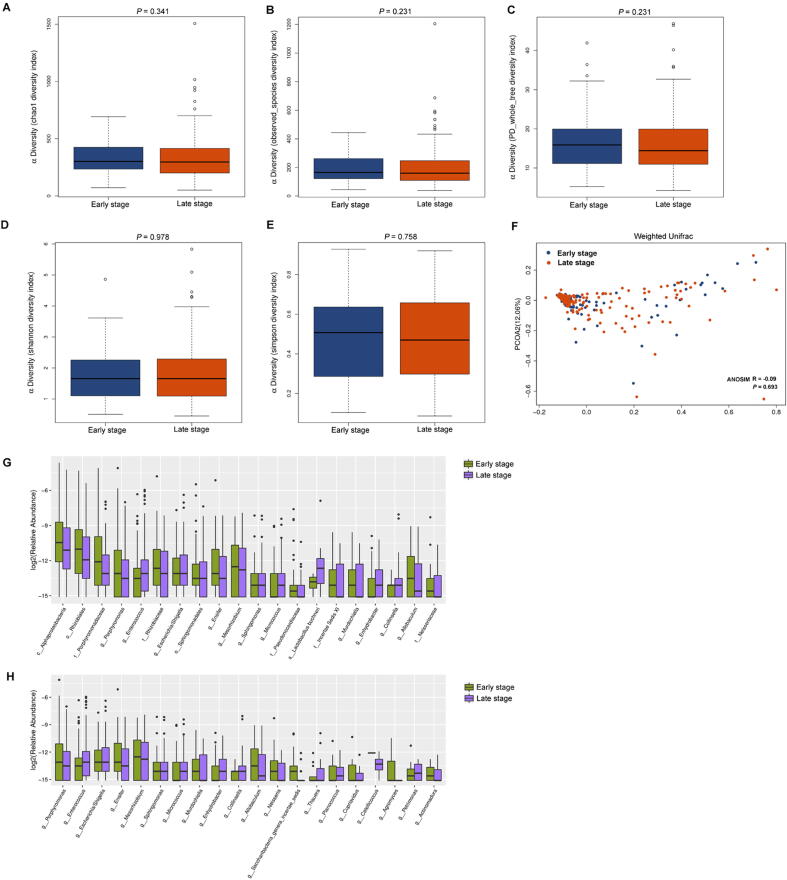
**Little difference was shown between early and late stages of pregnancy in microbial diversity, community structure and composition of the vaginal microbiome** A–E. Differences in the microbial diversities of the vaginal microbiome between early and late stages according to the Chao 1, observed species, PD whole tree, Shannon, and Simpson index. F. Weighted ANOSIM and PCoA based on the distance matrix of UniFrac dissimilarity of the vaginal microbiome of early and late pregnant stages. G. Relative abundance of the discriminating taxa in the vaginal microbiome between early and late pregnant stages at all levels. H. Relative abundance of the discriminating taxa in the vaginal microbiome of early and late pregnant stages at the genus level. The discriminating taxa were identified based on LefSe analysis with a threshold of (LDA scores (log10) > 2 and P < 0.05. The prefixes p_, c_, o_, f_, g_, and s_ represent phylum, class, order, family, genus, and species, respectively.

No significant differences in α and β diversities were observed (weighted UniFrac, ANOSIM *R* = −0.009, *P* = 0.693) (Figure S4A–F), nor did the bacterial taxa differ in relative abundance between the early (≤ 18 gestational weeks, *n* = 142) and late (27 ≤ gestational weeks < 42, *n* = 207) stages of pregnancy [*P* > 0.05 after false discovery rate (FDR) correction] (Figure S4G and H).

### The vaginal microbiome during pregnancy varies according to the presence of hypertensive disorders and abortion history

**Figure 2:**
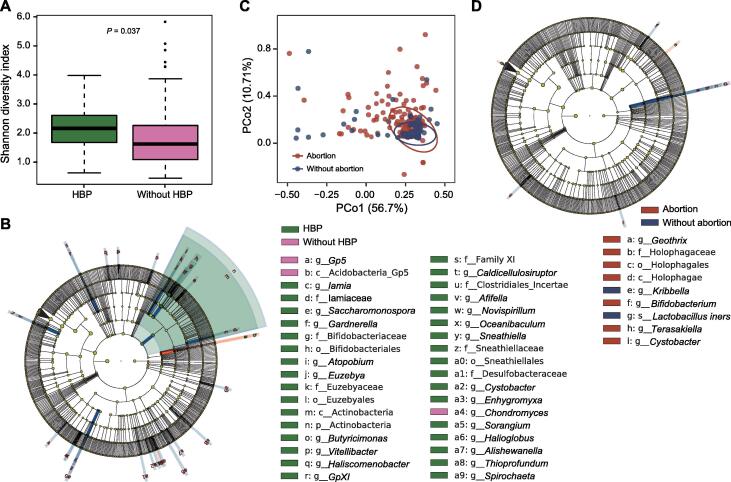
**The vaginal microbiome of pregnant women with a history of abortion or HBP changes during pregnancy** **A.** Alpha diversity of the vaginal microbiomes between pregnant women with and without HBP. Each box plot represents the median and IQR. The whiskers on each side of the box extend to the minimum or maximum value that is not an outlier. The circles beyond the whiskers represent outliers (> 1.5-fold IQR). **B.** Cladogram constructed using LEfSe indicates the phylogenetic distribution of the vaginal microbiome associated with pregnant women who presented with HBP. **C.** Weighted ANOSIM based on the distance matrix of UniFrac dissimilarity of the vaginal microbiome during pregnancy in women with or without an abortion history. The axes represent the two dimensions explaining the most considerable proportion of variance in the communities. Each dot represents a sample, and each circle indicates a 95% confidence interval. **D.** Cladogram constructed using LEfSe indicates the phylogenetic distribution of the vaginal microbiome associated with pregnant women who presented with a history of abortion. The LDA score (log_10_) > 2 and *P* < 0.05 are listed. HBP, high blood pressure/hypertensive disorder.

During pregnancy, the α diversity of the vaginal microbiome was significantly higher in pregnant women with hypertensive disorders (high blood pressure; HBP) than in those without HBP (*P* = 0.037, Figure 2A). The relative abundances of more than 30 bacterial taxa varied significantly between HBP and control groups, based on the results of linear discriminant analysis effect size (LEfSe) analysis (Figure 2B), and almost all such taxa were enriched, rather than depleted, in the case group. The presence of HBP was not associated with a reduction in the relative abundance of *Lactobacillus* but was accompanied by an increase in the proportions of genera, such as *Gardnerella*, *Atopobium*, and *Sneathia*. The results revealed that HBP might exert an impact on the vaginal microbiota during pregnancy to a certain extent, causing considerable changes in the diversity of bacterial species, part of which was consistent with that observed after delivery (Figure 1D).

A significant community structure difference was observed based on the weighted UniFrac distance (ANOSIM *R* = 0.054, *P* = 0.015) between pregnant women with and without a history of abortion (Figure 2C). The samples collected from women who did not have an abortion history showed more intensive changes, while those with a history of abortion were discrete. When screening for the taxonomic distinction between the two groups, only nine taxa were identified (Figure 2D). The relative abundance of *L. iners* was significantly lower in pregnant women with an abortion history. The clustering results indicate that the composition of the vaginal microbiome is also related to the abortion history. The microbiota of women who had not experienced a surgical abortion in the past was similar; on the contrary, abortion might have increased the heterogeneity between individuals, resulting in the establishment of completely different community structures. As there were more discriminating taxa that underwent significant changes, the impact of HBP on the vaginal microbiome seemed to be greater than that observed with the history of abortion. This may be partly attributable to the fact that HBP occurring during pregnancy exerts a greater impact on the maternal physiology, which would be immediately transferred to the microbiota, while a previous abortion history exerts an impact that is less remarkable.

**Supplementary Figure S5:**
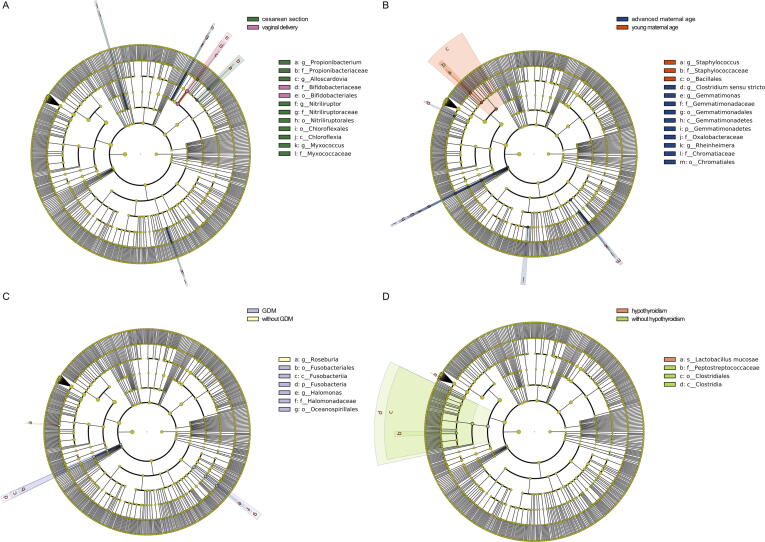
**LEfSe analysis showed the discriminant bacteria of vaginal microbiome during pregnancy corresponding to multiple complex factors** A. Different delivery mode. B. Advanced maternal age vs. young maternal age. C. With vs. without gestational diabetes mellitus. D. With vs. without hypothyroidism. The LDA scores (log10) > 2 and P < 0.05 are listed. The prefixes p_, c_, o_, f_, g_, and s_ represent phylum, class, order, family, genus, and species, respectively.

Although there were no significant differences in α and β diversities, several characteristic bacterial species corresponding to four factors, including the delivery mode, maternal age, gestational diabetes mellitus (GDM), and hypothyroidism, were found in the vaginal microbiome during pregnancy (Figure S5).

### The postpartum vaginal microbiome varies based on the delivery mode and maternal age

**Figure 3:**
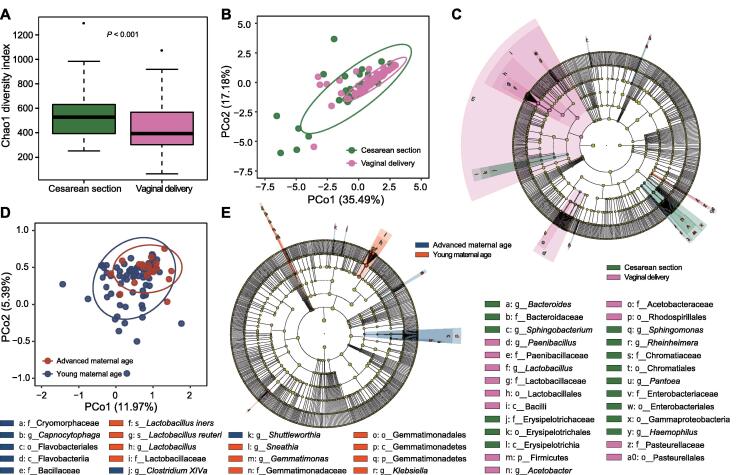
**The postpartum vaginal microbiome varies according to the delivery mode andmaternal age** **A.** Alpha diversity of the vaginal microbiomes between women who delivered vaginally and those who delivered via cesarean section in the postpartum period. Each box plot represents the median and IQR. The whiskers on each side of the box extend to the minimum or maximum value that is not an outlier. The circles beyond the whiskers represent outliers (> 1.5-fold IQR). **B.** Weighted ANOSIM based on the distance matrix of UniFrac dissimilarity of the vaginal microbiome in postpartum women who delivered vaginally or via cesarean section. The axes represent the two dimensions explaining the most considerable proportion of variance in the communities. Each dot represents a sample, and each circle indicates a 95% confidence interval. **C.** Cladogram constructed using LEfSe indicates the phylogenetic distribution of the vaginal microbiome associated with postpartum women who delivered vaginally or via cesarean section. **D.** Weighted ANOSIM based on the distance matrix of UniFrac dissimilarity of the vaginal microbiome in postpartum women who presented with an advanced age or young age. **E.** Cladogram constructed using LEfSe indicates the phylogenetic distribution of the vaginal microbiome associated with postpartum women who presented with an advanced age or young age. The LDA score (log_10_) > 2 and *P* < 0.05 are listed.

The postpartum vaginal microbiome showed a strong association with the delivery mode. After delivery, the microbial diversity of the vaginal microbiome in women who delivered via cesarean section was significantly higher than that in women who delivered vaginally (*P* < 0.001, Figure 3A). A significant community structure difference was also observed based on the weighted UniFrac distance between women who delivered via cesarean section and those who delivered vaginally, with the latter demonstrating a lower β diversity (ANOSIM *R* = 0.080, *P* = 0.034) (Figure 3B). It seemed that the vaginal microbiota of pregnant women who delivered vaginally was dominated by fewer microbial species after delivery, which also had a more similar community structure to each other. The discriminating taxa identified based on the LEfSe analysis results further reflected that *Lactobacillus*, which constitutes the resident microflora of the vagina of healthy women, was more abundant in the postpartum vagina of the pregnant women who delivered vaginally (Figure 3C).

**Supplementary Figure S6:**
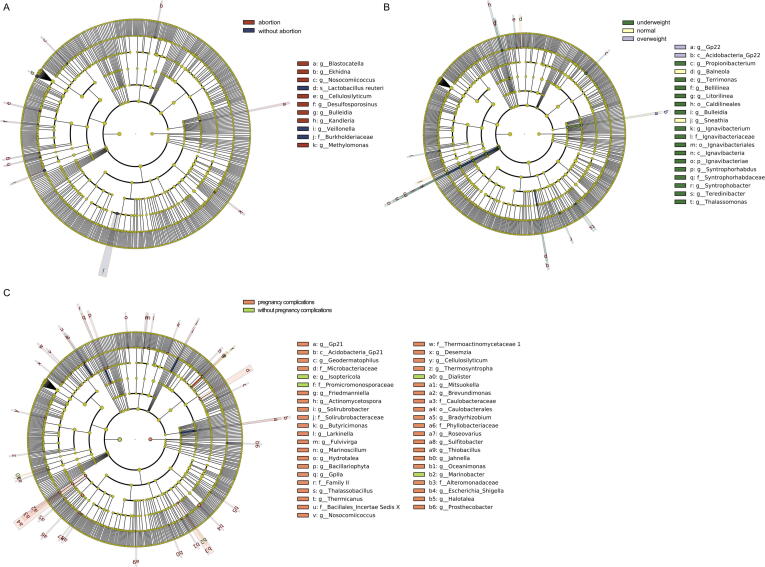
**LEfSe analysis showed the discriminant bacteria of vaginal microbiome in postpartum corresponding to multiple complex factors** A. Different body mass index before pregnancy. B. With vs. without abortion history. C. With vs. without pregnancy complications. The LDA scores (log10) > 2 and P < 0.05 are listed. The prefixes c_, o_, f_, g_, and s_ represent class, order, family, genus, and species, respectively.

We found that maternal age was another factor associated with variation in the postpartum vaginal microbiome. Samples collected from women of advanced and young ages were grouped into two distinct clusters (ANOSIM *R* = 0.149, *P* = 0.022) (Figure 3D), and the elderly formed a more consistent community structure. Based on the results of the LEfSe analysis, it was observed that the vaginal microbiome of the women with an advanced age lacked the *Lactobacillus* genus and its two species (*L. iners* and *L. reuteri*) after delivery (Figure 3E). In addition to the delivery mode and maternal age, marked shifts in the relative abundances of certain bacterial species were observed in the grouping of three other factors, namely abortion history, maternal pre-pregnancy weight status, and pregnancy complications (Figure S6).

### Identification of common key taxa accounting for the vaginal microbiome variation

**Supplementary Figure S7:**
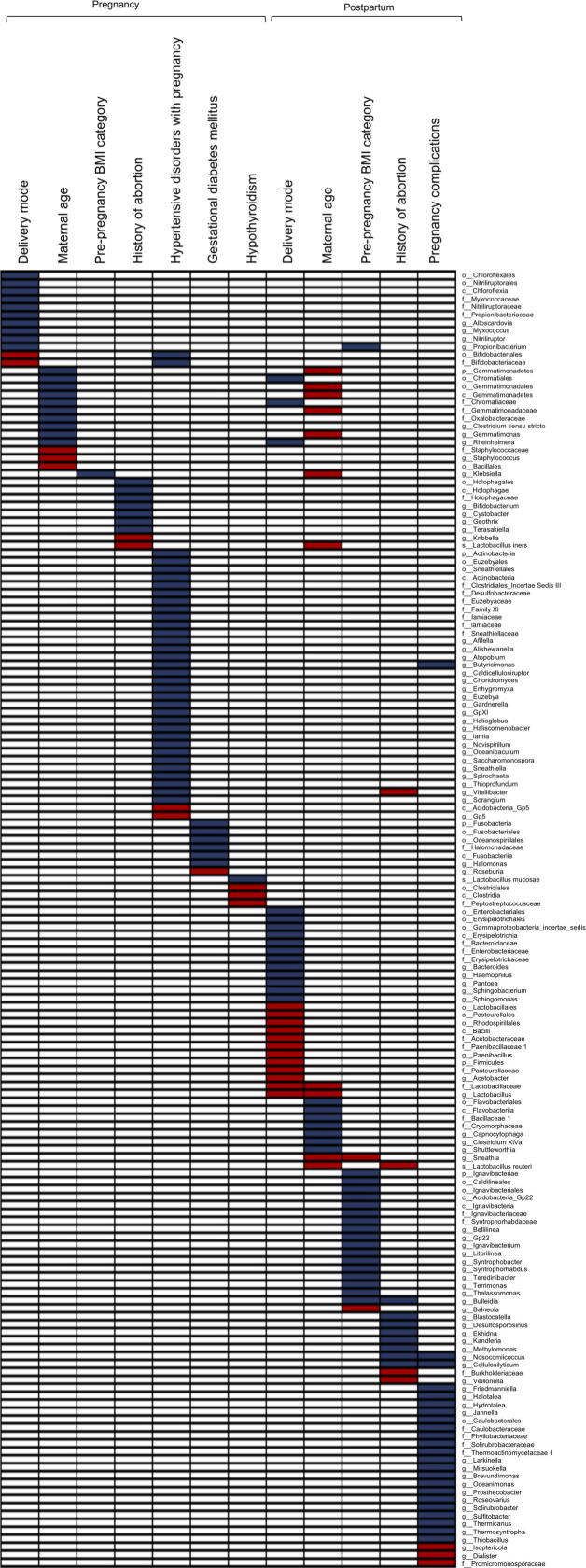
**Most discriminant bacteria of the vaginal microbiome were different under complex factors during pregnancy and postpartum** The relative abundance of 163 bacterial taxa, including 5 phyla, 12 classes, 22 orders, 35 families, 86 genera, and 3 species, varied significantly between groups during pregnancy and postpartum based on LefSe analysis. The LDA scores (log10) > 2 and P < 0.05 are listed. The boxes filled in blue and red color represent the discriminating taxa enriched and depleted in the vaginal microbiome of women who delivered cesarean, with advanced maternal age, overweight, hypertensive disorders, hypothyroidism or other pregnancy complications, and without abortion history or gestational diabetes mellitus during pregnancy and postpartum, respectively. The prefixes p_, c_, o_, f_, g_, and s_ represent phylum, class, order, family, genus, and species, respectively.

In total, the relative abundances of 163 bacterial taxa from the phylum to the genus levels varied significantly between groups during pregnancy and the postpartum period based on the results of LEfSe analysis (Figure S7). These bacterial taxa rarely overlapped across several factors, and a considerable proportion of the discriminating taxa were unique to each abnormal factor. Moreover, the discriminating taxa of the same factor showed no consistency between the prenatal and postpartum periods. Certain bacterial taxa associated with maternal age showed a completely opposite trend before and after delivery. The distinction between the discriminating taxa and the abundance divergence of the same taxa reflected substantial vaginal microbial community differences during pregnancy and the postpartum period, as well as their response to various abnormal factors.

**Figure 4:**
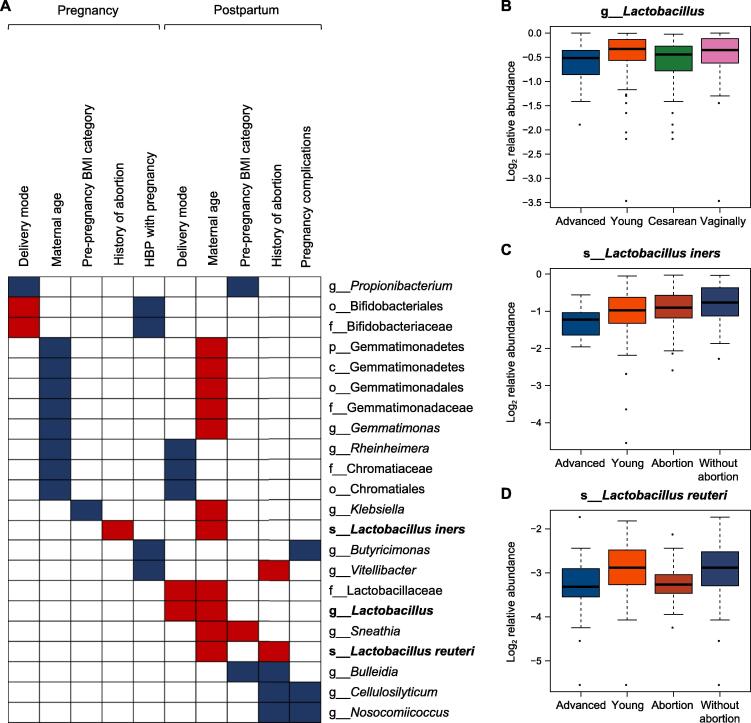
**The abundances of specific vaginal bacteria change in multiple groups during pregnancy and the postpartum period** **A.** The relative abundances of the 22 bacterial taxa simultaneously varied significantly in two of the ten comparisons during pregnancy and the postpartum period, based on the results of LEfSe analysis. The discriminating taxa were identified using a threshold of LDA score (log_10_) > 2 and *P* < 0.05. The blue and red boxes represent the discriminating taxa enriched and depleted in the vaginal microbiome of women who delivered via cesarean section, with an advanced maternal age, who were overweight, with HBP or other pregnancy complications, and with an abortion history during pregnancy and the postpartum period, respectively. **B.** The relative abundance of the *Lactobacillus* genus was depleted in postpartum women who delivered via cesarean section or with an advanced maternal age. **C.** The relative abundance of *Lactobacillus iners* was depleted in pregnant women with an abortion history and in postpartum women with an advanced maternal age. **D.** The relative abundance of *Lactobacillus reuteri* was depleted in postpartum women with an abortion history or with an advanced maternal age. BMI, body mass index.

Several common taxa accounting for the variation of the vaginal microbiome could be identified from the 163 discriminating taxa. The relative abundances of 22 bacterial taxa were simultaneously altered in two of the ten comparisons (Figure 4A). Among them, 13 taxa showed identical trends in two comparisons, and significant differences were consistently recorded for eight genera, namely *Propionibacterium*, *Rheinheimera*, *Butyricimonas*, *Lactobacillus*, *Sneathia*, *Bulleidia*, *Cellulosilyticum*, and *Nosocomiicoccus*. Common taxa were more likely associated with the delivery mode and maternal age; particularly, the postpartum microbiome was associated with maternal age. Additionally, the relative abundance of the *Lactobacillus* genus decreased in postpartum women who delivered via cesarean section or with an advanced maternal age (Figure 4B). The relative abundance of *L. iners* was lower in pregnant women with an abortion history and in postpartum women with an advanced maternal age (Figure 4C). The relative abundance of *L. reuteri* was depleted in postpartum women with an abortion history or with an advanced maternal age (Figure 4D). These results suggest that vaginal delivery, a young maternal age, and no history of abortion may be more appropriate for the growth of *Lactobacillus* in the maternal vagina, which will be beneficial for the health and well-being of women. Few vaginal microbes showed the same response to different abnormal factors and might be more susceptible to their influence; therefore, these bacterial species, together with the maternal age, warrant additional studies for validation.

### Variations in the CSTs are associated with multiple factors during pregnancy and the postpartum period

**Figure 5:**
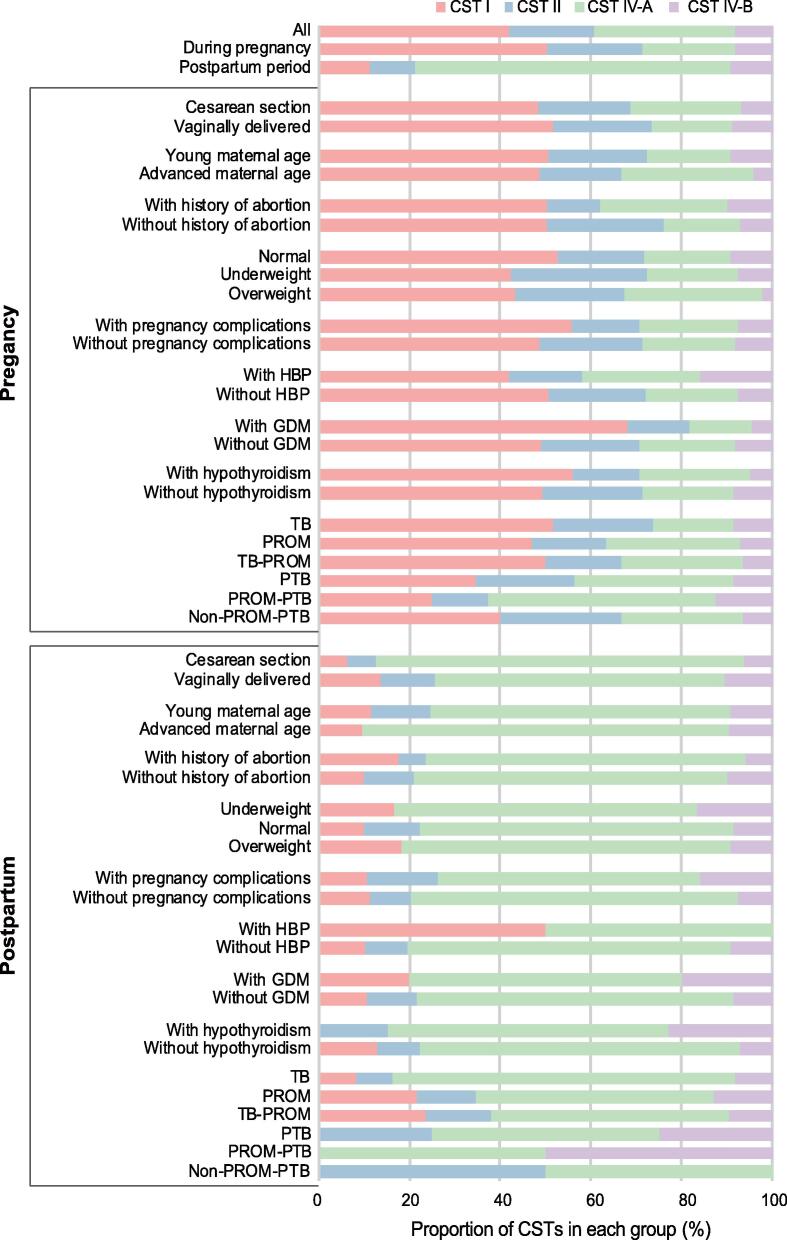
**The prevalence of CSTs in the vaginal microbiome is different depending on different maternal factors** The percentage of each CST in each group is represented by using one color. The top three bars show the mean of the CST percentage in all samples, in the samples during pregnancy, and in the samples during the postpartum period, respectively. TB denotes the pregnant woman with a term delivery; TB-PROM denotes the occurrence of PROM along with TB; PROM-PTB indicates PTB complicated by PROM; and non-PROM-PTB denotes PTB without PROM. TB, term birth; PTB, perterm birth; PROM, premature rupture of membranes; GDM, gestational diabetes mellitus.

To facilitate the comparison of the relationships between the vaginal microbiome and the several factors in different periods, we examined the distribution of CSTs (Figure 5; Table S3). The most striking difference before and after delivery was that the CST I and CST IV-A presented with the highest proportions, respectively. In the vaginal microbiome during pregnancy, the ratios of CST IV-A in the cesarean section, advanced maternal age, with abortion history, overweight, HBP, and hypothyroidism groups were remarkably higher than those in the corresponding groups. Notably, the difference is remarkable in CST composition between pregnant women who undergo normal (term) delivery and those who have PTB and PROM. The term delivery (term birth, TB) group during pregnancy showed a lower CST IV-A/CST I ratio (0.35), and this ratio increased to 0.53 if PROM occurred, even though there was no premature birth (TB-PROM). Additionally, a high CST IV-A/CST I ratio (1.00) was observed in the group of pregnant women with PTB, and this ratio was particularly high (2.00) during pregnancy in women with PROM complicated by PTB (PROM-PTB). After delivery, the higher abundance of CST IV-A in the vaginal microbiome was further exacerbated, except in women with HBP. The divergence between TB and PTB did not disappear, and CST I was not detected in both PROM-PTB and PTB.

**Supplementary Figure S8:**
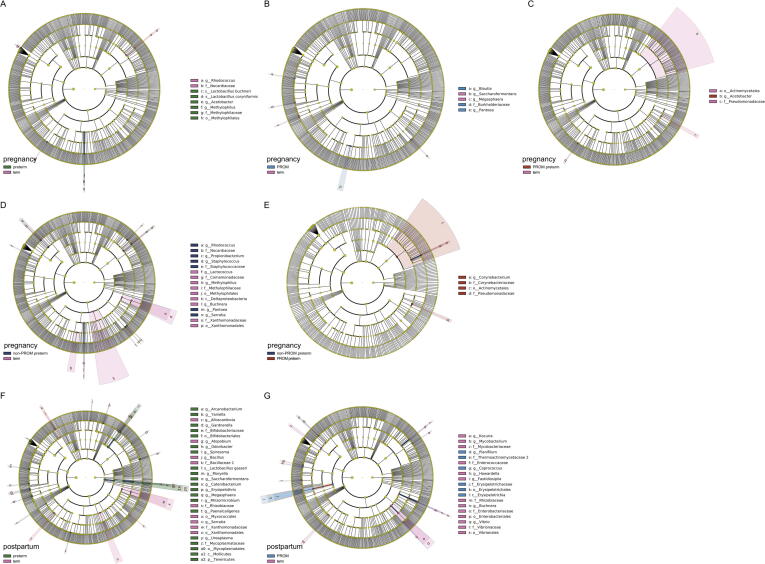
**Preterm birth and PROM were accompanied by some bacteria enrichment in the vaginal microbiome** Based on LefSe analysis, the relative abundances of 68 bacterial taxa varied significantly between groups. The LDA scores (log10) > 2 and P < 0.05 are listed. A. Preterm vs. term group during pregnancy. B. PROM vs. term group during pregnancy. C. PROM related preterm vs. term group during pregnancy. D. Non-PROM related preterm vs. term group during pregnancy. E. PROM related preterm vs. non-PROM related preterm during pregnancy. F. Preterm vs. term group in postpartum. G. PROM vs. term group in postpartum. The prefixes p_, c_, o_, f_, g_, and s_ represent phylum, class, order, family, genus, and species, respectively.

To further investigate the connections between the vaginal microbiome and the PTB and PROM, the microbial compositions were compared between different groups during pregnancy and the postpartum period. The relative abundances of 68 bacterial taxa, including 1 phylum, 3 classes, 9 orders, 18 families, 34 genera, and 3 species, varied significantly between groups (Figure S8A–G). During pregnancy, the relative abundances of 6 bacterial taxa, including *Lactobacillus buchneri* and *Lactobacillus coryniformis*, and 3 bacterial taxa were increased in pregnant women with PTB and PROM, respectively, compared to those with TB (Figure S8A and B). When we stratified the pregnant women with PTB based on their PROM statuses, 3 bacterial taxa were associated with PROM-PTB (Figure S8C) and 16 bacterial taxa were associated with PTB without PROM (non-PROM-PTB) (Figure S8D). Among the pregnant women who delivered preterm infants, the relative abundances of 4 bacterial taxa were significantly higher in those with PROM-PTB than in those with non-PROM-PTB (Figure S8E). These discriminant bacterial species showing significant changes in prenatal abundance in the vaginal microbiome may serve as candidate biomarkers for predicting PTB. In the postpartum period, 20 bacterial taxa, including *L. gasseri*, were more abundant in the PTB group, while 9 bacterial taxa, including *Bacillus*, were more abundant in the TB group (Figure S8F). Significant differences in the relative abundances of the 19 bacterial taxa were also recorded between postpartum women with and without PROM (Figure S8G).

## Discussion

Both cross-sectional and longitudinal studies have reported that the vaginal microbiome during pregnancy is less diverse and more stable than that during the postpartum period [20]. Chinese women are not represented in such studies appropriately. To this end, this study characterized the vaginal microbiome of the largest cohort of Chinese women examined. Consistent with previous studies in women of African, Hispanic, or European populations [10], [20], we also found that the diversity and the composition of the vaginal microbial communities were relatively stable at the early and late time points during pregnancy and markedly changed during the postpartum period, exhibiting less *Lactobacillus* dominance in the Chinese population. Furthermore, we found that the vaginal microbiome composition changed according to the abortion history and HBP during pregnancy, and the delivery mode and maternal age during the postpartum period.

Identification of key taxa within the diverse vaginal microbiome is of immense importance because they may pose differing risks of adverse health outcomes in pregnant women. We identified a series of significant bacterial taxa that differed significantly in relative abundance according to the delivery mode, maternal age, history of abortion, and pregnancy complications. Notably, we also found several key taxa that differed significantly in relative abundance in two of ten comparisons during pregnancy and the postpartum period. Considering that these key taxa have been consistently shown to be associated with the health of women in previous studies [6], [11], they are of particular importance for future studies. Other taxa, such as *Propionibacterium*, which is enriched in cesarean section newborns [21], *Butyricimonas*, which is enriched in patients with autism spectrum disorder [22] and depleted in patients with histamine intolerance [23] and thyroid cancer [24], and *Bulleidia*, which is more frequent in patients with esophageal squamous cell carcinoma [25], also merit additional investigation.

CST is widely used in the studies of vaginal microbiome to deal with inter-subject and/or intra-subject variability of women [26]. A comparison of this new dataset with existing datasets derived from Caucasian women and women from other countries revealed that the prevalence of CSTs might differ across populations. Using a large cohort size of 396 North American women from four ethnic groups, Ravel et al. [18] established five CSTs. Huang et al. [27] identified four CSTs in 34 Chinese women during different pregnancy stages. Considering that there is a distinct lack of information regarding the CSTs present in Chinese pregnant women, our results obtained from a large population of Chinese women contribute to a more comprehensive understanding of the CSTs in Chinese pregnant women. Similar to the findings reported by Huang et al. [27], we also identified the following four CSTs previously described: I (41.9%), III (18.7%), IV-A (31.1%), and IV-B (8.3%); however, CST II and CST V were not found in our study population. The possible reasons for the lack of CST II and CST V in Chinese women and the differences between them and Caucasian women may be attributed to their living environment or ethnicity.

Unexpectedly, we found that the changes in the vaginal microbiome community were also related to GDM, which has not been reported in either Chinese or Caucasian populations. Compared with healthy pregnant women, women with GDM demonstrated a higher proportion of CST I during pregnancy, while the proportion of non-*Lactobacillus*-dominated CST IV was lower. After delivery, the proportion of CST I in women with GDM remained higher than that in healthy women. However, unlike that observed during pregnancy, the proportion of CST IV-B, which contains increased proportion of *Gardnerella* and is generally considered to be unfavorable to vaginal health [18], increased remarkably in postpartum women with GDM. These results suggest that GDM may be more harmful to the postpartum vaginal microbiome and health than to the prenatal microbiome, and the postpartum health of such a female population warrants emphasis and consideration.

In the present study, we have presented a study design with the challenge of mixed, and even discordant, findings in some studies linking the vaginal microbiome to PTB. Consistent with the findings reported by Romero [4], we found no differences in the frequency of the observed CSTs between women with a term delivery and those with a preterm delivery. However, compared with their results, which did not demonstrate key taxa differing in relative abundance [4], we found that the bacterial taxa were significantly different between women with PROM-PTB and those with non-PROM-PTB, indicating that different types of PTB were related with specific bacterial taxa. A few contributing taxa, such as *Gardnerella*, *Ureaplasma*, and *Megasphaera*, could be considered for developing predictive models in Chinese populations. We can replicate the previously reported associations between *Gardnerella*, *Ureaplasma,* or *Megasphaera* and clinically heterogeneous PTB in cohorts of individuals belonging predominantly to an African descent in the postpartum stage [5], [7]. The previously hypothesized associations between a lower *Lactobacillus* abundance and PTB in cohorts of individuals belonging predominantly to an African descent, and between a higher *L. iners* abundance and PTB in cohorts of individuals belonging predominantly to a European descent, were not replicated in our study [5], [6], [7]. The differences between previous studies and our results strongly suggest that the PTB–microbiome associations may be race-dependent. Further population-specific studies are warranted to assess the impact of the association between the vaginal microbiome and PTB and to identify the presence of population-specific key taxa.

Although the present study represents the most extensive examination of the vaginal microbiome of Chinese pregnant women conducted thus far, in reviewing our results, several limitations should be considered. First, this was a cross-sectional study in which samples were obtained at a single time point. Prospective longitudinal studies are necessary to confirm our results. Second, studies using metagenomic sequencing are warranted in the future to provide more detailed information about the function and changes in the vaginal microbiome. Additionally, the absence of a concurrent analysis of host factors may be explain the variable conclusions obtained [28].

## Conclusion

This is the first study to characterize the vaginal microbiome of a Chinese cohort of such a size. We identified measurable differences in the vaginal microbiome of Chinese pregnant women in terms of the delivery mode, maternal age, history of abortion, and HBP, with possible consequences in both the short- and long-term health statuses of women. We also demonstrated that the PTB–microbiome associations were population-dependent and revealed new insights into the ethnic and biogeographical effects on the association between PTB and the vaginal microbiome. With the realization that our understanding of the microbiome is as good as the diversity of the individuals sampled, this dataset will provide valuable information for future research and contribute to a more comprehensive understanding of the correlation between bacterial communities and Chinese pregnant women.

## Materials and methods

### Patients and samples

All participants provided informed consent. Information regarding the demographic characteristics, medical history, clinical manifestations, and laboratory test results were prospectively collected using a standard questionnaire. For each participant, a cotton swab was used to collect the discharge from the posterior vagina. The samples were stored at −20 °C upon collection. All samples were stored at −80 °C for 4 h until metagenomic DNA extraction.

### Processing of the microbial samples

Microbial DNA extraction from the vaginal swabs and sterile water was conducted using a DNA extraction kit (Catalog No. 51704, Qiagen, Hilden, Germany) according to the manufacturer’s instructions. The DNA concentration and purity were measured using Qubit (ThermoFisher Scientific, Waltham, MA). DNA samples were subjected to amplification using polymerase chain reaction (PCR) and a pair of barcoded primers (341F: CCTAYGGGRBGCASCAG; 806R: GGACTACNNGGGTATCTAAT) targeting the V3–V4 region of the 16S rRNA gene. Each PCR reaction was conducted in a 30-µl reaction system with 15 µl of the Phusion High-Fidelity PCR Master Mix (Catalog No. M0531S, New England Biolabs, Ipswich, MA), 0.2 µM forward and reverse primers, and approximately 10 ng of the DNA template. Negative extraction controls and blank controls were included by using sterile water as a substitute for the PCR template. The same volume of 1× loading buffer (containing SYBR green) was mixed with the PCR products, and electrophoresis was performed using a 2% agarose gel for detection. Samples with a bright main strip in the 400–450 bp range were selected for further experiments. The PCR products were pooled at equimolar ratios. The mixture was then purified using the GeneJET Gel Extraction Kit (Catalog No. K0691, ThermoFisher Scientific). Sequencing libraries were constructed using the NEB Next Ultra DNA Library Prep Kit for Illumina (Catalog No. E7370L, New England Biolabs) following the manufacturer’s recommendations. Library quality was assessed using the Agilent Bioanalyzer 2100 system (Agilent Technologies, Palo Alto, CA). Finally, the library was sequenced using the Illumina HiSeq platform (Illumina, San Diego, CA), and 250 bp paired-end reads were generated.

### Bioinformatics and statistical analyses

Paired-end reads were merged into long sequences based on the overlaps between reads1 and reads2 by using FLASH [29]. Merged sequences then were analyzed using QIIME (v1.9.1) software package [30]. First, sequences were filtered by QIIME quality filters. Then we used “pick_de_novo_otus.py” to pick OTUs in addition to generating an OTU table. Sequences with a ≥ 97% similarity were assigned to the same OTUs. A representative sequence was picked for each OTU and the Ribosomal Database Project (RDP) database was used to generate taxonomic information for each representative sequence.

To compute α diversity, the OTU table was rarified and five metrics including Chao1, observed species, PD whole tree (Faith’s Phylogenetic Diversity, which adds up all the branch lengths of the phylogenetic tree as a measure of diversity), Shannon, and Simpson were calculated. Rarefaction curves were generated based on these metrics. Both weighted and unweighted Unifrac distances were calculated for principal coordinate analysis (PCoA). The pairwise dissimilarity between the microbial community structures was assessed using Bray-Curtis distance at the OTU level as described before [31]. The difference in microbial markers was measured using Mann-Whitney rank test and LEfSe. When multiple hypothesis tests were performed simultaneously, *P* values were corrected using Benjamini and Hochberg’s FDR. For the comparative analysis, only the genera and species with the abundance of > 1% and > 0.2%, respectively, in at least one of the samples were included.

The clustering of CSTs was done using complete linkage hierarchical clustering with five clusters as described by Ravel and his colleagues [18], [20]. CST I, CST II, CST III, and CST V were predominated with *L. crispatus*, *L. gasseri*, *L. iners*, and *L. jensenii*, respectively*.* CST IV was defined as lacking *Lactobacillus* spp. and comprising a diverse set of strict and facultative anaerobes, and was further split into CST IV-A and CST IV-B.

## Ethical statement

The study was performed with the approval of the Ethical Committee of Beijing Institute of Microbiology and Epidemiology, China and conducted according to the principles expressed in the Declaration of Helsinki. All participants provided informed consent.

## Data availability

The sequencing data have been deposited in the Genome Sequence Archive [32] at the National Genomics Data Center, Beijing Institute of Gemonics, Chinese Academy of Sciences / China National Center for Bioinformation (GSA: CRA002692), and are publicly accessible at https://ngdc.cncb.ac.cn/gsa.

## CRediT author statement

**Xiaoai Zhang:** Conceptualization, Methodology, Investigation, Writing - original draft, Writing - review & editing. **Qingzhi Zhai:** Resources, Data curation, Supervision, Writing - original draft. **Jinfeng Wang:** Conceptualization, Methodology, Software, Writing - original draft, Writing - review & editing. **Xiuling Ma:** Software, Visualization. **Bo Xing:** Resources, Data curation. **Hang Fan:** Software. **Zhiying Gao:** Resources. **Fangqing Zhao:** Conceptualization, Writing - original draft, Writing - review & editing, Project administration, Funding acquisition. **Wei Liu:** Conceptualization, Writing - original draft, Writing - review & editing, Project administration, Funding acquisition. All authors have read and approved the final manuscript.

## Competing interests

The authors have declared no competing interests.
