# Supplementary material for: Variation of the Vaginal Microbiome During and After Pregnancy in Chinese Women
Source: Genomics Proteomics Bioinformatics. 2022 Jan 28;20(2):322–33. doi: 10.1016/j.gpb.2021.08.013 (PMC9684158; doi:10.1016/j.gpb.2021.08.013)
Supplement: Supplementary Table S1 [file mmc1.docx]

**Table S1 Characteristics of pregnant women included in the study**

| **Group** | **Total**  **(*n* = 454)** | **During pregnancy**  **(*n* = 356)** | **Postpartum**  **(*n* = 98)** | ***P* value ^*^** |
| --- | --- | --- | --- | --- |
| Delivery mode |  |  |  | 0.042 |
| Cesarean section | 189 | 157 | 32 |  |
| Vaginally delivered | 265 | 199 | 66 |  |
| Maternal age (years old) |  |  |  | 0.794 |
| Young maternal age (< 35) | 361 | 284 | 77 |  |
| Advanced maternal age (≥ 35) | 93 | 72 | 21 |  |
| Abortion history |  |  |  | 0.002 |
| Abortion | 138 | 121 | 17 |  |
| Without abortion | 316 | 235 | 81 |  |
| Pre-pregnancy body mass index |  |  |  | 0.270 |
| Underweight (BMI < 18.5) | 46 | 40 | 6 |  |
| Normal (18.5 ≤BMI < 25) | 351 | 270 | 81 |  |
| Overweight (BMI ≥ 25) | 57 | 46 | 11 |  |
| Pregnancy complications |  |  |  | 0.357 |
| Hypertensive disorders with pregnancy | 21 | 19 | 2 |  |
| Gestational diabetes mellitus | 27 | 22 | 5 |  |
| Hypothyroidism | 126 | 41 | 13 |  |
| Adverse pregnancy outcomes |  |  |  |  |
| Preterm Rupture of Membranes | 91 | 68 | 23 | 0.983 |
| Term-PROM | 81 | 60 | 21 |  |
| Preterm-PROM | 10 | 8 | 2 |  |
| Clinically heterogeneous preterm birth | 27 | 23 | 4 | 0.613 |
| PROM-preterm birth | 10 | 8 | 2 |  |
| Non-PROM-preterm birth | 17 | 15 | 2 |  |

*Note*: PROM, Preterm Rupture of Membranes. ^*^, the *P* value was calculated by the *χ*^2^ test or Fisher's exact test.
