# Supplementary material for: Variation of the Vaginal Microbiome During and After Pregnancy in Chinese Women
Source: Genomics Proteomics Bioinformatics. 2022 Jan 28;20(2):322–33. doi: 10.1016/j.gpb.2021.08.013 (PMC9684158; doi:10.1016/j.gpb.2021.08.013)
Supplement: Supplementary Table S3 [file mmc3.docx]

**Table S3 Distribution of community state types according to delivery mode, age, abortion, BMI, pregnancy and delivery cxiuzhengons**

|  | **CST Ⅰ** | **CST Ⅲ** | **CST Ⅳ-A** | **CST Ⅳ-B** | ***P* value** | ***χ*^2^** |
| --- | --- | --- | --- | --- | --- | --- |
| **All** | 190 (41.9) | 85 (18.7) | 141 (31.1) | 38 (8.3) |  |  |
| **Gestational stage** |  |  |  |  | < 0.001 | 92.08 |
| **During pregnancy** | 179 (50.3) | 75 (21.1) | 73 (20.5) | 29 (8.1) |  |  |
| **Delivery mode** |  |  |  |  | 0.46 | 2.58 |
| Cesarean section | 76 (48.4) | 32 (20.4） | 38 (24.2) | 11 (7.0) |  |  |
| Vaginally delivered | 103 (51.8) | 43 (21.6） | 35 (17.6) | 18 (9.0) |  |  |
| **Maternal age (years old)** |  |  |  |  |  |  |
| Young maternal age (< 35) | 144 (50.7) | 62 (21.8) | 52 (18.3) | 26 (9.2) | 0.14 | 5.49 |
| Advanced maternal age (≥ 35) | 35 (48.6) | 13 (18.1) | 21 (29.2) | 3 (4.1) |  |  |
| **Abortion history** |  |  |  |  | 0.003 | 13.71 |
| Abortion | 61 (50.4) | 14 (11.6) | 34 (28.1) | 12 (9.9) |  |  |
| Without abortion | 118 (50.2) | 61 (26.0) | 39 (16.6) | 17 (7.2) |  |  |
| **Pre-pregnancy BMI category** |  |  |  |  |  |  |
| Normal | 142 (52.66) | 52 (19.3) | 51 (18.9) | 25 (9.2) |  |  |
| Underweight | 17 (42.5) | 12 (30.0) | 8 (20.0) | 3 (7.5) | 0.43 | 2.70 |
| Overweight | 20 (43.5) | 11 (23.9) | 14 (30.4) | 1 (2.2) | 0.11 | 6.01 |
| **Pregnancy complications** |  |  |  |  |  |  |
| Hypertensive disorders with pregnancy |  |  |  |  | 0.51 | 2.30 |
| With | 8 (42.1) | 3 (15.8) | 5 (26.3) | 3 (15.8) |  |  |
| Without | 171 (50.7) | 72 (21.4) | 68 (20.2) | 26 (7.7) |  |  |
| Gestational diabetes mellitus |  |  |  |  | 0.39 | 3.02 |
| With | 15 (68.2) | 3 (13.6) | 3 (13.6) | 1 (4.4) |  |  |
| Without | 164 (49.1) | 72 (21.6) | 70 (21.0) | 28 (8.3) |  |  |
| Hypothyroidism |  |  |  |  | 2.17 | 0.54 |
| With | 23 (56.1) | 6 (14.6) | 10 (24.4) | 2 (4.9) |  |  |
| Without | 156 (49.5) | 69 (21.9) | 63 (20.0) | 27 (8.6) |  |  |
| **Delivery complications** |  |  |  |  |  |  |
| Term & not PROM | 141 (51.7) | 60 (22.0) | 49 (17.9) | 23 (8.4) |  |  |
| PROM | 32 (47.1) | 11 (16.2) | 20 (29.4) | 5 (7.3) | 0.19 | 4.72 |
| Term-PROM | 30 (50.0) | 10 (16.7) | 16 (26.7) | 4 (6.6) | 0.43 | 2.79 |
| Preterm-PROM | 2 (25.0) | 1 (12.5) | 4 (50.0) | 1 (12.5) | 0.12 | 5.79 |
| Preterm | 8 (34.8) | 5 (21.7) | 8 (34.8) | 2 (8.7) | 0.23 | 4.32 |
| PROM-preterm | 2 (25.0) | 1 (12.5) | 4 (50.0) | 1 (12.5) | 0.12 | 5.79 |
| Non-PROM-preterm | 6 (40.0) | 4 (26.7) | 4 (26.7) | 1 (6.6) | 0.76 | 1.16 |
| **Postpartum period** | 11 (11.2) | 10 (10.2) | 68 (69.4) | 9 (9.2) |  |  |
| **Delivery mode** |  |  |  |  | 0.36 | 3.18 |
| Cesarean section | 2 (6.2) | 2 (6.2) | 26 (81.4) | 2 (6.2) |  |  |
| Vaginally delivered | 9 (13.6) | 8 (12.1) | 42 (63.6) | 7 (10.7) |  |  |
| **Maternal age (years old)** |  |  |  |  |  |  |
| Young maternal age (< 35) | 9 (11.7) | 10 (13.0) | 51 (66.2) | 7 (9.1) | 0.35 | 3.31 |
| Advanced maternal age (≥ 35) | 2 (9.5) | 0 (0) | 17 (81.0) | 2 (9.5) |  |  |
| **Abortion history** |  |  |  |  | 0.71 | 1.38 |
| Abortion | 3 (17.6) | 1 (5.9) | 12 (70.6) | 1 (5.9) |  |  |
| Without abortion | 8 (9.9) | 9 (11.1) | 56 (69.1) | 8 (9.9) |  |  |
| **Pre-pregnancy BMI category** |  |  |  |  |  |  |
| Underweight | 1 (16.7) | 0 (0) | 4 (66.6) | 1 (16.7) | 0.71 | 1.39 |
| Normal | 8 (9.9) | 10 (12.3) | 56 (69.2) | 7 (8.6) |  |  |
| Overweight | 2 (18.2) | 0 (0) | 8 (72.7) | 1 (9.1) | 0.57 | 1.99 |
| **Pregnancy complications** |  |  |  |  |  |  |
| Hypertensive disorders with pregnancy |  |  |  |  | 0.36 | 3.23 |
| With | 1 (50.0) | 0 (0) | 1 (50.0) | 0 (0) |  |  |
| Without | 10 (10.4) | 9 (9.4) | 68 (70.8) | 9 (9.4) |  |  |
| Gestational diabetes mellitus |  |  |  |  | 0.65 | 1.64 |
| With | 1 (20.0) | 0 (0) | 3 (60.0) | 1 (20.0) |  |  |
| Without | 10 (10.8) | 10 (10.8) | 65 (69.9) | 8 (8.5) |  |  |
| Hypothyroidism |  |  |  |  | 0.15 | 5.36 |
| With | 0 (0) | 2 (15.4) | 8 (61.5) | 3 (23.1) |  |  |
| Without | 11 (12.9) | 8 (9.4) | 60 (70.6) | 6 (7.1) |  |  |

*Note*: CST community state type based on ward HCA of species data. The numbers in brackets indicate percentages. BMI, Body Mass Index.
